# Supplementary material for: Oncoprotein SET-associated transcription factor ZBTB11 triggers lung cancer metastasis
Source: Nat Commun. 2024 Feb 14;15:1362. doi: 10.1038/s41467-024-45585-5 (PMC10867109; doi:10.1038/s41467-024-45585-5)
Supplement: Supplementary file 1 — Supplementary Information [file 41467_2024_45585_MOESM1_ESM.pdf]

Supplementary Information

Oncoprotein SET-associated Transcription Factor ZBTB11 Triggers Lung Cancer Metastasis

Authors:

Wenbin Xu[1, #], Han Yao[1, #], Zhen Wu[1], Xiaojun Yan[1], Zishan Jiao[1], Yajing Liu[1], Meng Zhang[1], Donglai Wang[1, *]

[#] These authors contributed equally to this work.

[*] Corresponding author (dwang@ibms.pumc.edu.cn)

Affiliations:

1. State Key Laboratory of Common Mechanism Research for Major Diseases & Department of Medical Genetics, Institute of Basic Medical Sciences & School of Basic Medicine, Chinese Academy of Medical Sciences & Peking Union Medical College, Beijing 100005, China

This file contains Supplementary Figures 1-11

Supplementary Figure 1

a

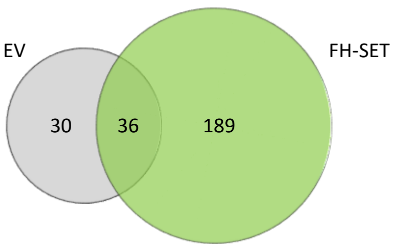

b

Identified SET-specific binding proteins by MS (Top 15)

Rank	Protein	Total peptides	Unique peptides
1	TRIM28	52	21
2	PRRC2A	30	25
3	HMGA1	17	6
4	**SET**	17	4
5	RIF1	16	13
6	HSPA5	14	10
7	SF3B2	12	11
8	NPM1	10	9
9	SF3A1	9	8
10	**ZBTB11**	9	7
11	HNRNPC	8	4
12	HNRNPA2B1	7	6
13	HCFC1	7	6
14	GATAD2A	7	6
15	SNRPB	7	4

c

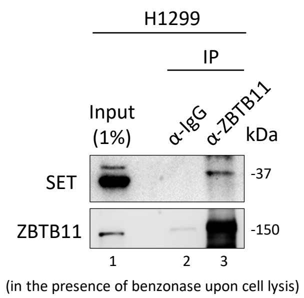

d

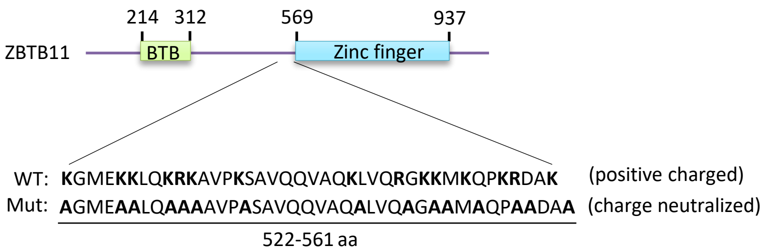

e

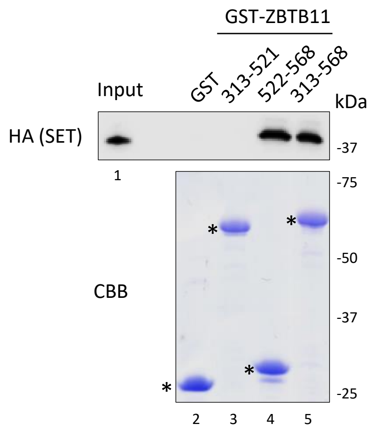

f

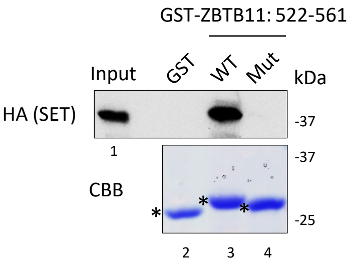

g

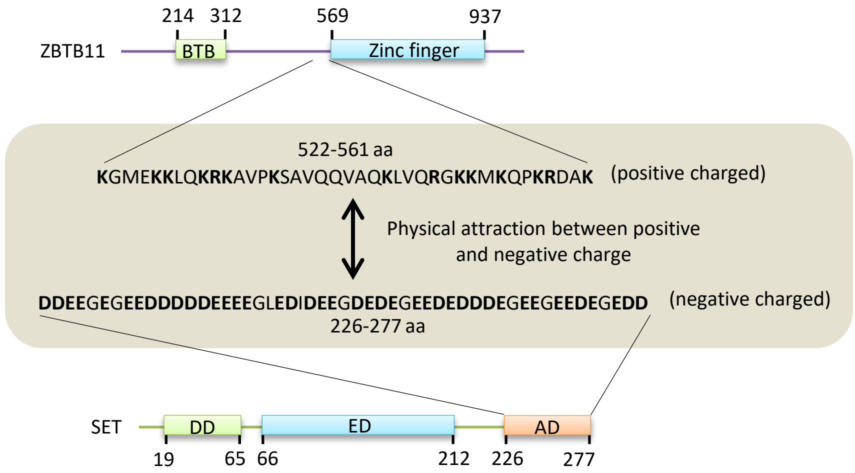

Supplementary Figure 1 Mechanistic analysis of the SET-ZBTB11 interaction. (**a**) Venn diagram of the proteins identified by TAP-MS from H1299-EV or H1299-FH-SET stable cells. (**b**) List of the top 15 SET-specific binding proteins based on TAP-MS analysis. (**c**) Co-IP-WB analysis of the interaction between endogenous SET and ZBTB11 in H1299 cells. DNA was degraded by treatment with benzonase (250 U/ml) upon cell lysis. (**d**) Schematic diagram of the K/R-rich region (522-561 aa) of ZBTB11. Mut: a mutant K/R-rich region where all lysine (K) and arginine (R) residues were replaced with alanine (A) residues. (**e**) *In vitro* pull-down analysis of the domain(s) of ZBTB11 responsible for mediating their physical interaction with purified SET. (**f**) *In vitro* pull-down analysis of the direct interaction between purified SET and the wild-type or mutant K/R-rich region of ZBTB11. (**g**) Schematic diagram of the "charge effect" as a basis of the SET-ZBTB11 physical interaction, where the negative charge within the acidic domain (AD) of SET attracts the positive charge within the K/R-rich region of ZBTB11. Source data are provided as a Source Data file. * indicates GST or GST-fusion protein.

Supplementary Figure 2

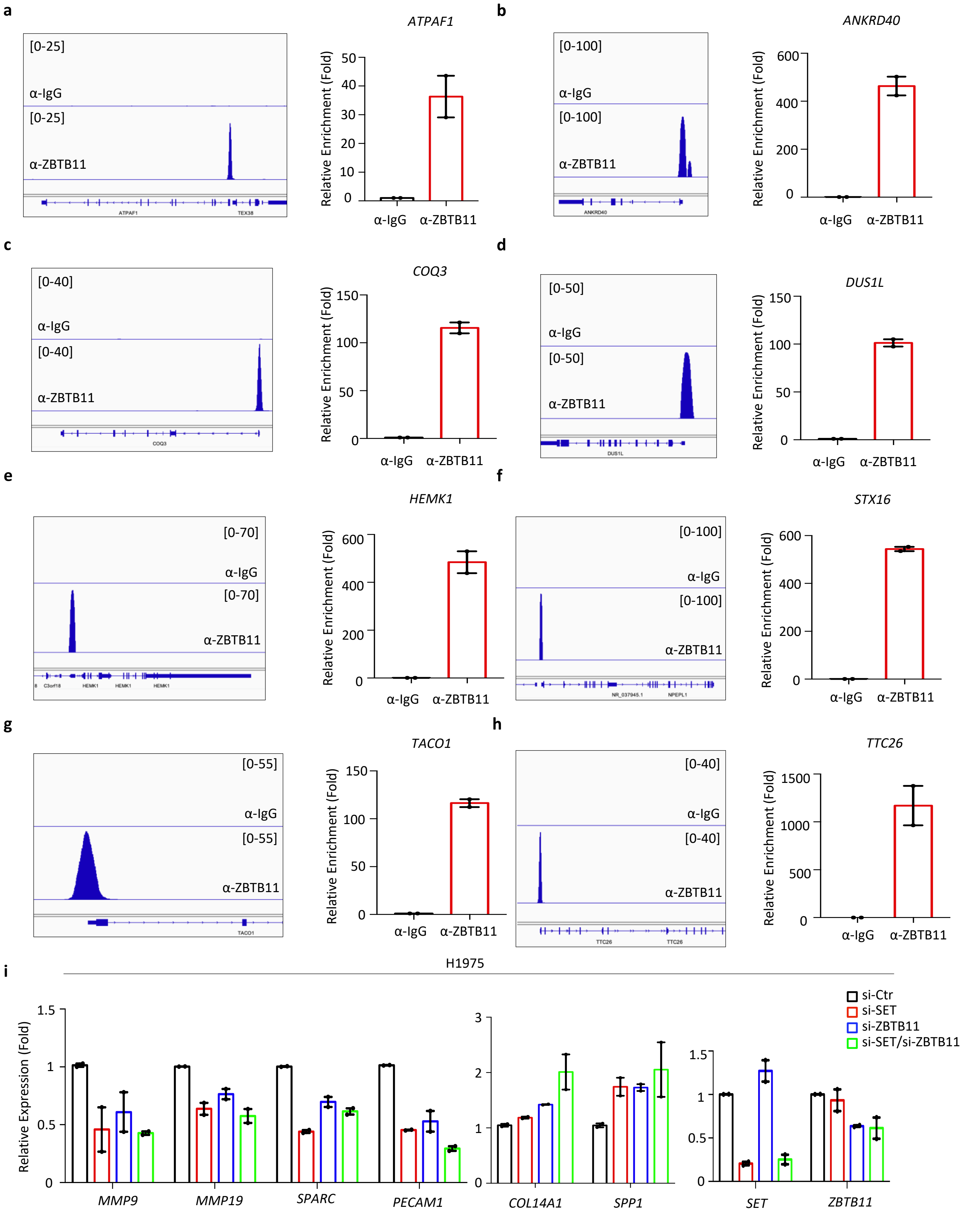

Supplementary Figure 2 Validation of ZBTB11 ChIP-seq analysis and SET/ZBTB11 coregulated target genes. (**a-h**) ZBTB11-binding peaks and ChIP–qPCR analysis of ZBTB11 enrichment on potential DNA-binding elements of genes, including *ATPAF1* (**a**), *ANKRD40* (**b**), *COQ3* (**c**), *DUS1L* (**d**), *HEMK1* (**e**), *STX16* (**f**), *TACO1* (**g**), and *TTC26* (**h**), in H1299 cells. Data were shown as mean ± S.E.M., n=2 experimental replicates. (**i**) RT–qPCR analysis of the representative genes involved in extracellular matrix organization in H1975 cells depleted with or without SET and/or ZBTB11 for 96 hrs. Data were shown as mean ± S.E.M., n=2 experimental replicates. Source data are provided as a Source Data file.

Supplementary Figure 3

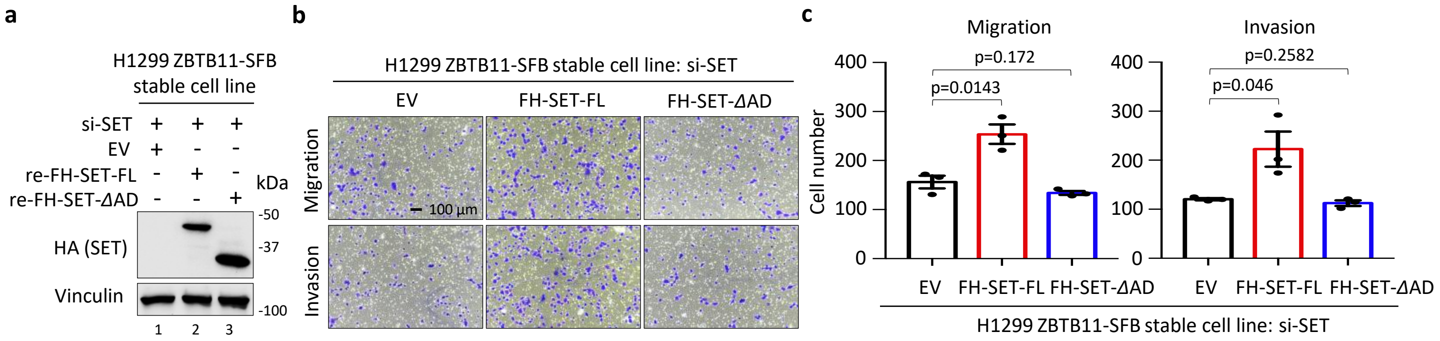

Supplementary Figure 3 Acidic domain is critical for SET-mediated metastatic regulation of lung cancer cells with ZBTB11 overexpression. (**a**) WB analysis of H1299-ZBTB11-SFB cells depleted of endogenous SET by siRNA and re-expressed with RNAi-resistant ectopic full-length or acidic domain-truncated SET (re-FH-SET-FL or re-FH-SET-⊿AD). (**b**-**c**) Cell migration and invasion of endogenous SET-depleted H1299-ZBTB11-SFB cells re-expressed with re-FH-SET-FL or re-FH-SET-⊿AD. Data were shown as the mean ± S.E.M., n=3 biologically independent samples, two-sided t-test. Source data are provided as a Source Data file.

Supplementary Figure 4

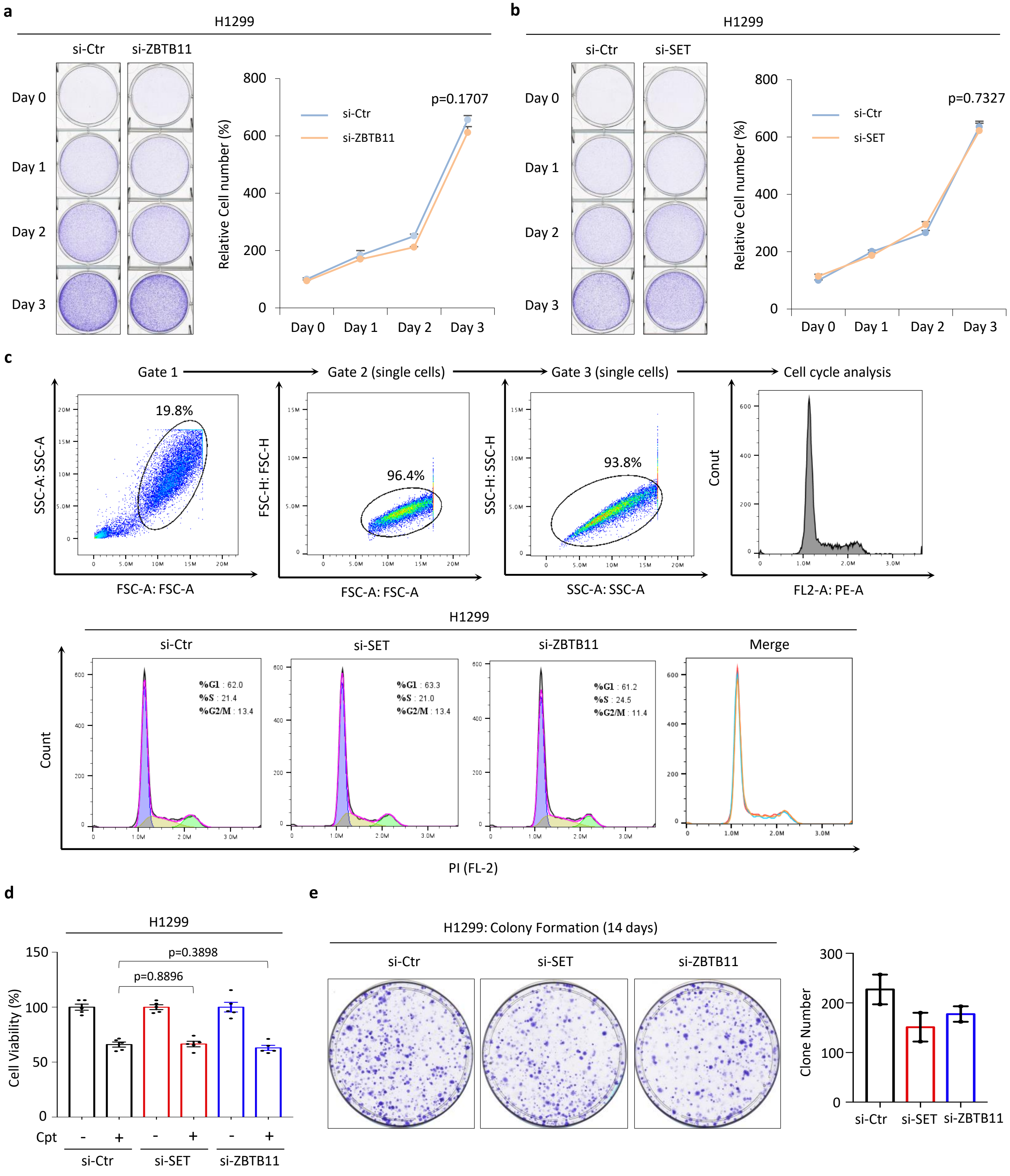

Supplementary Figure 4 Knockdown of ZBTB11 or SET had no obvious effect on cell growth, cell cycle progression, cell viability in response to DNA damage, and colony formation. (**a-b**) Cell proliferation of H1299 cells with or without ZBTB11 depletion (**a**) or SET depletion (**b**). Data were shown as the mean±S.E.M., n=3 biologically independent samples. The p-values were determined by two-sided t-test. (**c**) Cell cycle analysis of H1299 cells transiently transfected with control siRNA (si-Ctr) or siRNA targeting SET (si-SET) or ZBTB11 (si-ZBTB11). The gating strategy was shown on the upper panel. (**d**) The viability of H1299 cells depleted with or without SET or ZBTB11 in response to 1 μM camptothecin (Cpt) treatment for 24 hrs. Data were shown as the mean±S.E.M., n=5 biologically independent samples. The p-values were determined by two-sided t-test. (**e**) Colony formation of H1299 cells depleted with or without SET or ZBTB11. Data were shown as the mean±S.E.M., n=2 biologically independent samples. Source data are provided as a Source Data file.

Supplementary Figure 5

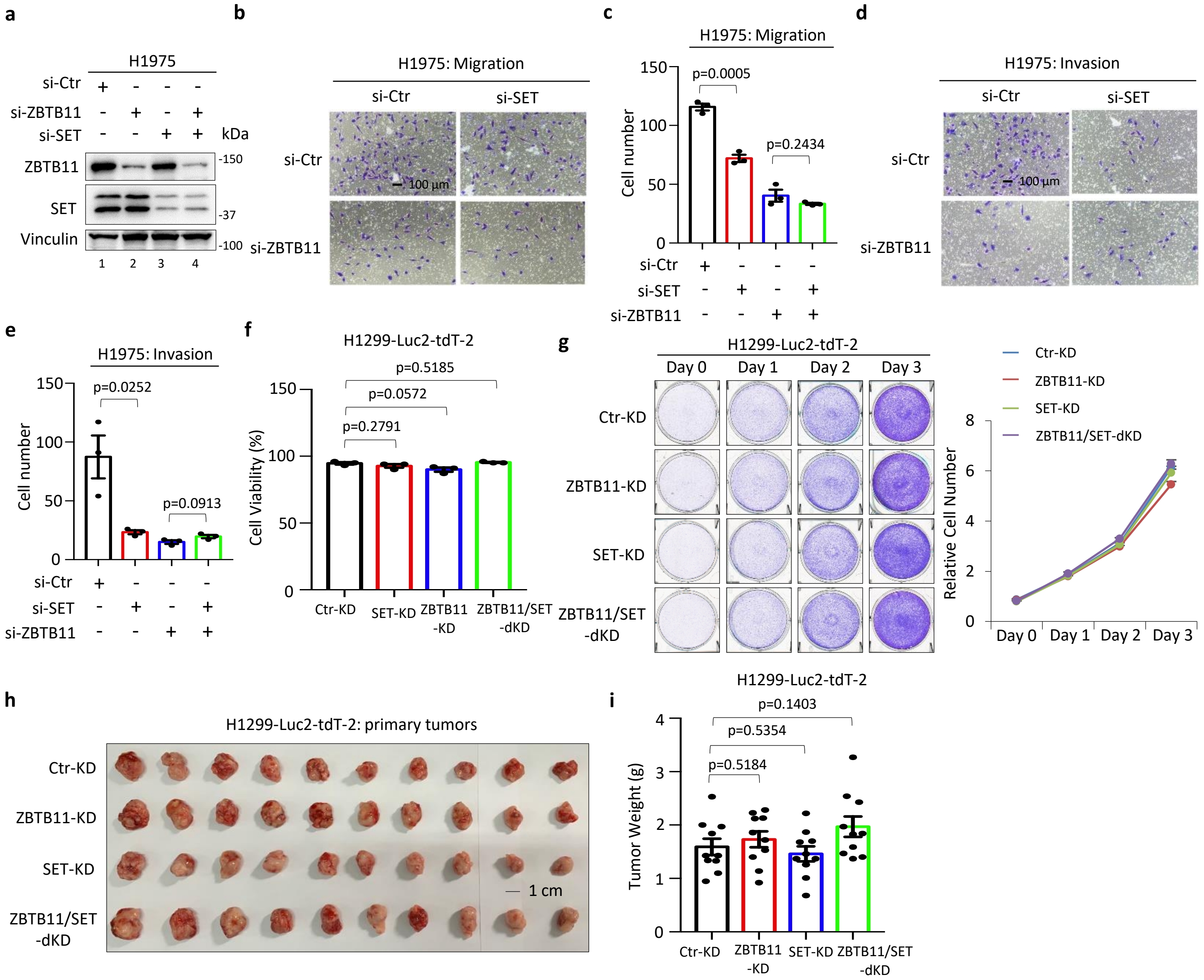

Supplementary Figure 5 The roles of ZBTB11 and SET in cell migration, invasion, proliferation and tumorigenesis. (**a**) WB analysis of SET and ZBTB11 knockdown efficiency in H1975 cells transiently transfected with control siRNA (si-Ctr) or siRNA against SET (si-SET) or ZBTB11 (si-ZBTB11) for 96 hrs. (**b-e**) Cell migration (**b** and **c**) or invasion (**d** and **e**) assays of H1975 cells upon SET and/or ZBTB11 depletion. Data were shown as the mean±S.E.M., n=3 biologically independent samples. The p-values were determined by two-sided t-test. (**f**) Viability of H1299-Luc2-tdT-2 cells with or without stable SET/ZBTB11 knockdown. Data were shown as the mean±S.E.M., n=3 biologically independent samples. The p-values were determined by two-sided t-test. (**g**) Proliferation of H1299-Luc2-tdT-2 cells with or without stable SET/ZBTB11 knockdown. Data were shown as the mean±S.E.M., n=3 biologically independent samples. (**h**) Primary tumors from a xenograft mouse model in which H1299-Luc2-tdT-2 cells with or without SET/ZBTB11 knockdown were subcutaneously inoculated into the flanks of immunodeficient B-NDG (NSG) mice. (**i**) Quantitative analysis of the tumor weight from (**h**). Data were shown as the mean±S.E.M., n=10 tumors per group. The p-values were determined by two-sided t-test. Source data are provided as a Source Data file.

Supplementary Figure 6

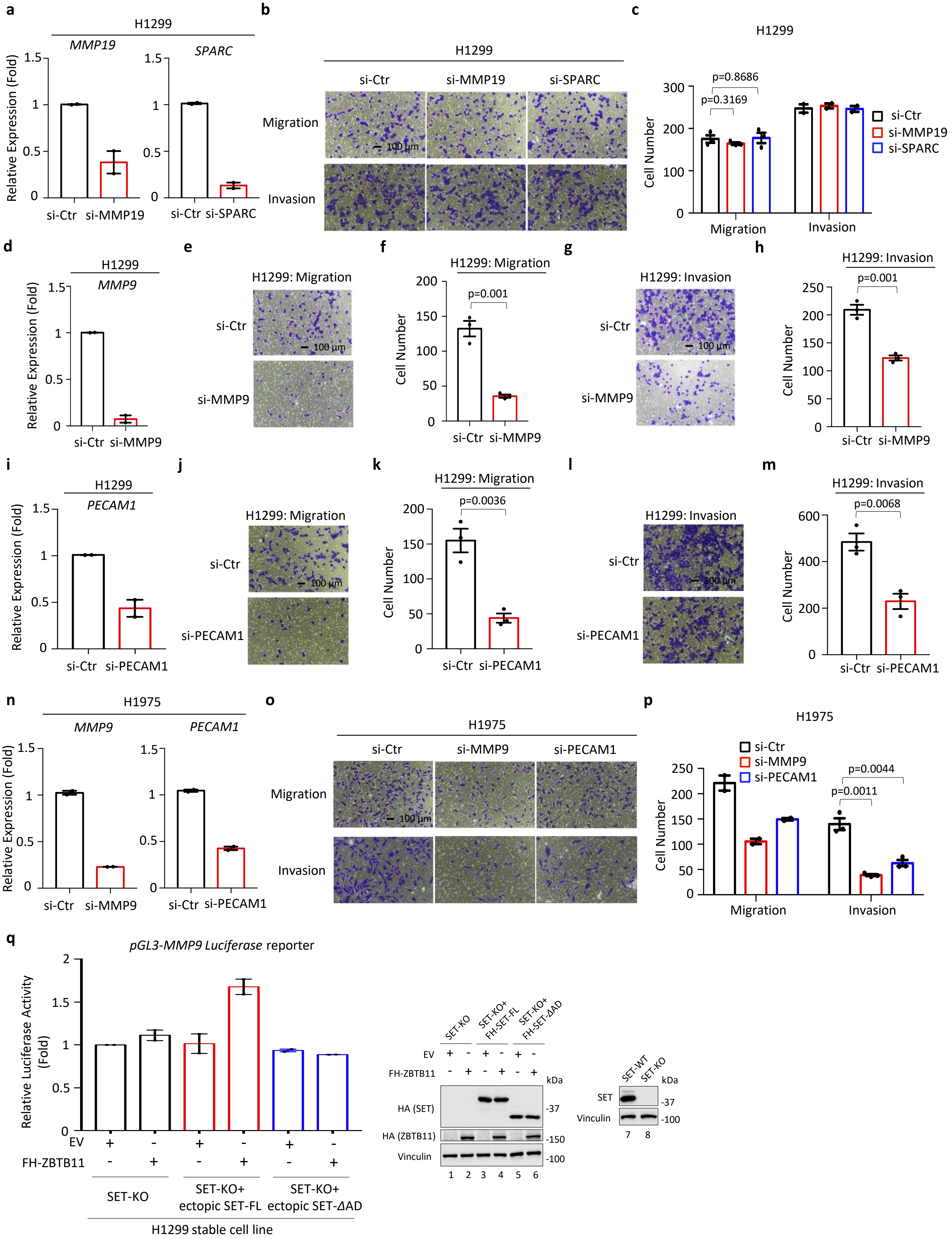

Supplementary Figure 6 Potential downstream effectors of SET-ZBTB11 complex in metastatic regulation. (**a**) RT–qPCR analysis of the knockdown efficiency of *MMP19* or *SPARC* in H1299 cells (mean ± S.E.M., n=2 experimental replicates). (**b-c**) Cell migration and invasion of H1299 cells upon MMP19 or SPARC depletion (mean ± S.E.M., n=3 biologically independent samples for migration, n=2 biologically independent samples for invasion, two-sided t-test). (**d**) RT–qPCR analysis of MMP9 knockdown efficiency in H1299 cells transiently transfected with si-Ctr or siRNA against *MMP9* (mean ± S.E.M., n=2 experimental replicates). (**e-h**) Cell migration (**e** and **f**) and invasion (**g** and **h**) assays of H1299 cells upon MMP9 depletion (mean ± S.E.M., n=3 biologically independent samples, two-sided t-test). (**i**) RT–qPCR analysis of the knockdown efficiency of *PECAM1* in H1299 cells (mean ± S.E.M., n=2 experimental replicates). (**j-m**) Cell migration (**j** and **k**) or invasion (**l** and **m**) of H1299 cells upon PECAM1 depletion (mean ± S.E.M., n=3 biologically independent samples, two-sided t-test). (**n**) RT–qPCR analysis of the knockdown efficiency of *MMP9* or *PECAM1* in H1975 cells (mean ± S.E.M., n=2 experimental replicates). (**o-p**) Cell migration and invasion of H1975 cells upon MMP9 or PECAM1 depletion. (mean ± S.E.M., n=2 biologically independent samples for migration and n=3 biologically independent samples for invasion, two-sided t-test). (**q**) Luciferase assays of SET/ZBTB11-driven transcriptional regulation of *MMP9*. The luciferase reporter containing the ZBTB11-binding element of the *MMP9* loci and renilla control were cotransfected with or without the ZBTB11-expressing vector into edited H1299 cells, as indicated, for 24 hrs (mean ± S.E.M., n=2 experimental replicates). SET-KO: H1299 cells with SET knockout (KO) by the CRISPR/Cas9 technique; SET-KO+ectopic SET-FL: SET KO H1299 cells stably re-expressing ectopic full-length SET; SET-KO+ectopic SET-*Δ*AD: SET KO H1299 cells stably re-expressing ectopic acidic domain-truncated SET. SET knockout, as well as the re-expression of ectopic SET, was validated by WB. Source data are provided as a Source Data file.

Supplementary Figure 7

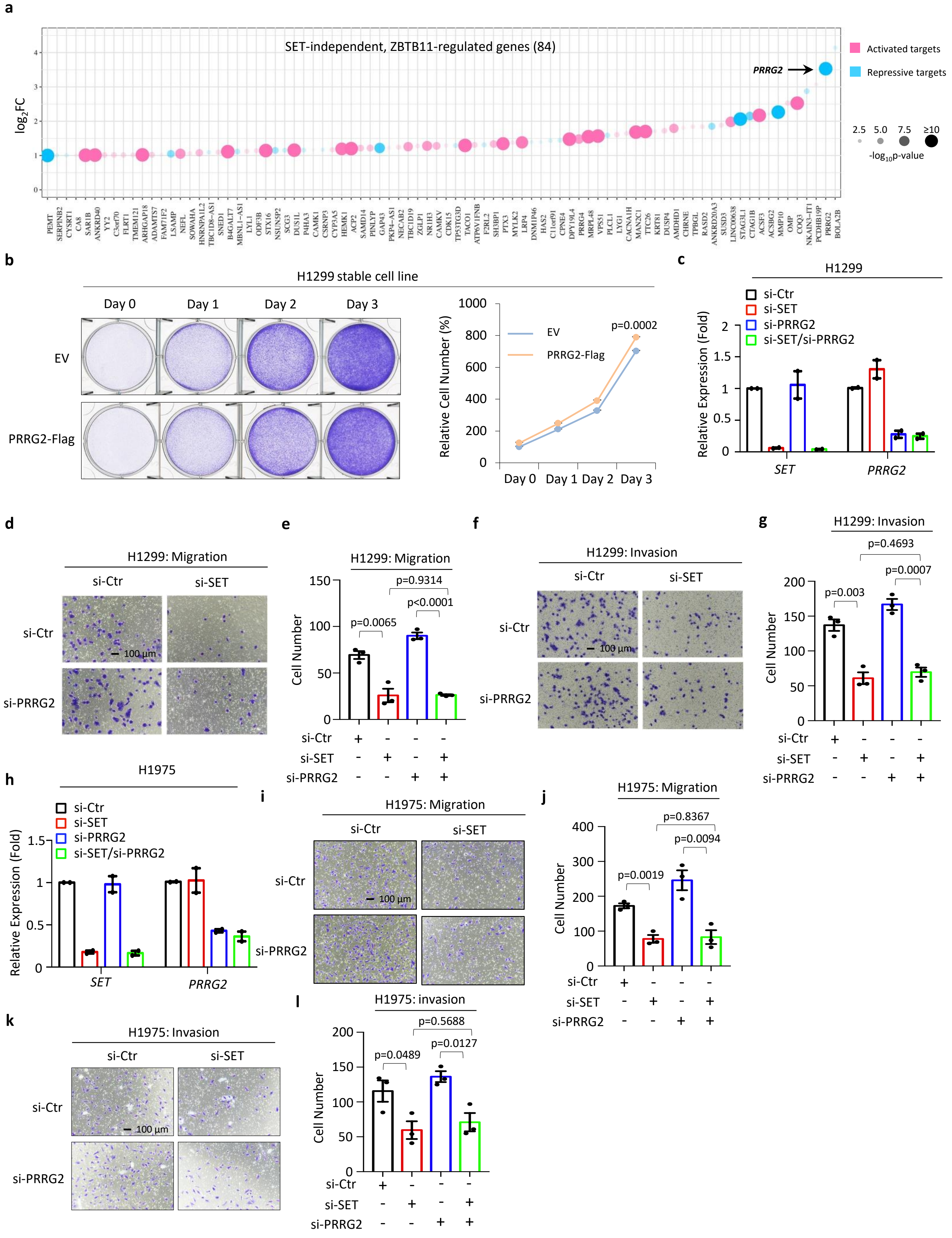

Supplementary Figure 7 Characterization of PRRG2 as a potential downstream effector of ZBTB11 in metastatic regulation. (**a**) Differentially expressed genes (84) regulated by ZBTB11 but not by SET identified through RNA-seq. The genes were ranked by |log2 (fold change)|. The bubble size was positively correlated with the statistical significance. The p-values were determined by Wald test. (**b**) Cell proliferation of H1299-EV or H1299-PRRG2-Flag stable cells for 3 days. Data were shown as the mean±S.E.M., n=3 biologically independent samples. The p-value was determined by two-sided t-test. (**c**) RT–qPCR analysis of *SET* or *PRRG2* expression in H1299 cells depleted with or without SET or PRRG2. Data were shown as the mean±S.E.M., n=2 experimental replicates). (**d-g**) Cell migration (**d** and **e**) or invasion (**f** and **g**) assays of H1299 cells with SET or PRRG2 depletion alone or together, as indicated. Data were shown as the mean±S.E.M., n=3 biologically independent samples. The p-values were determined by two-sided t-test. (**h**) RT–qPCR analysis of *SET* or *PRRG2* expression in H1975 cells depleted with or without SET or PRRG2. Data were shown as the mean±S.E.M., n=2 experimental replicates). (**i-l**) Cell migration (**i** and **j**) or invasion (**k** and **l**) assays of H1975 cells with SET or PRRG2 depletion alone or together, as indicated. Data were shown as the mean±S.E.M., n=3 biologically independent samples. The p-value was determined by two-sided t-test. Source data are provided as a Source Data file.

Supplementary Figure 8

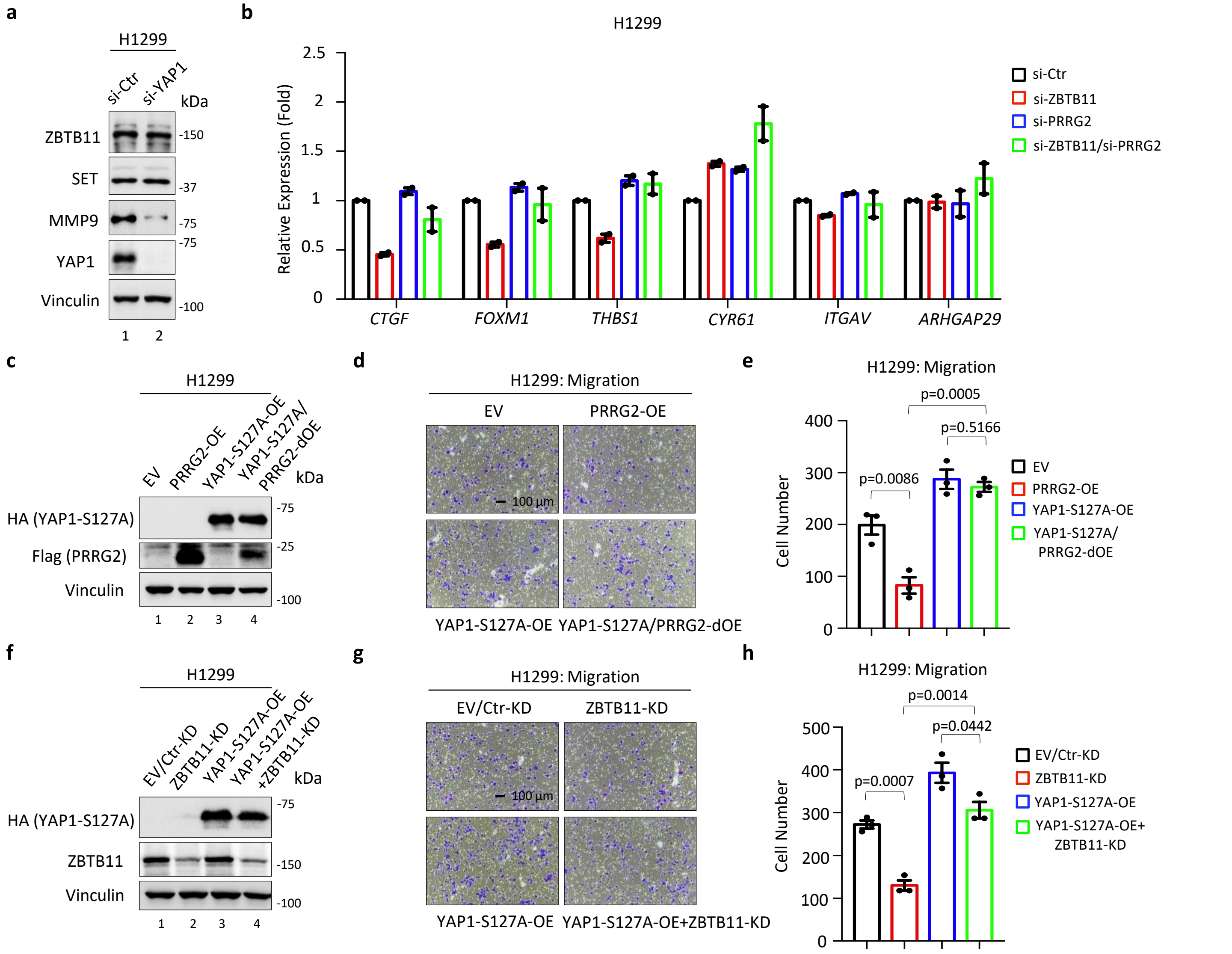

Supplementary Figure 8 The roles of YAP1 in ZBTB11-PRRG2 axis-mediated metastatic regulation. (**a**) WB analysis of ZBTB11, SET and MMP9 expression in H1299 cells depleted with or without YAP1. (**b**) RT–qPCR analysis of a group of metastasis-related target genes of *YAP1* in H1299 cells with ZBTB11 and/or PRRG2 knockdown. Data were shown as the mean±S.E.M., n=2 experimental replicates. The knockdown efficiency of ZBTB11 and PRRG2 was shown in Figure 6f. (**c**) WB analysis of PRRG2 and YAP1 in H1299 cells stably expressing PRRG2 (PRRG2-OE) and/or the YAP1-S127A mutant (YAP1-S127A-OE), as indicated. (**d-e**) Cell migration of H1299 cells with PRRG2-OE and/or YAP1-S127A-OE. Data were shown as the mean±S.E.M., n=3 biologically independent samples. The p-value was determined by two-sided t-test. (**f**) WB analysis of ZBTB11 and YAP1 in H1299 cells stably expressing shRNA targeting ZBTB11 (ZBTB11-KD) and/or stably expressing the YAP1-S127A mutant construct (YAP1-S127A-OE), as indicated. (**g-h**) Cell migration of H1299 cells with ZBTB11-KD and/or YAP1-S127A-OE. Data were shown as the mean±S.E.M., n=3 biologically independent samples. The p-value was determined by two-sided t-test. Source data are provided as a Source Data file.

Supplementary Figure 9

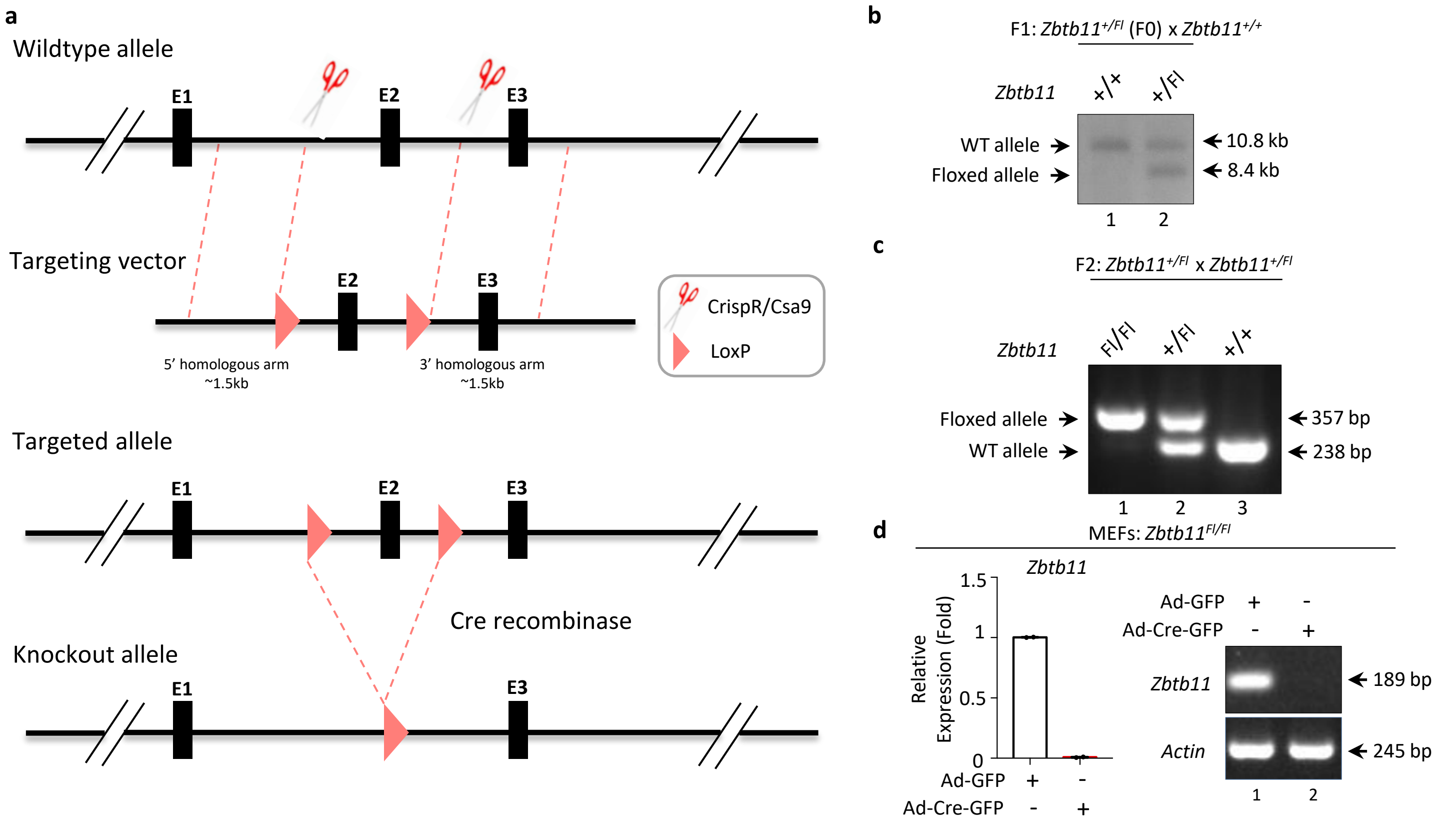

e

Zbtb11$^{+/-}$ X *Zbtb11*$^{+/-}$

↓

Offspring (Total 21 mice)

Genotype	*Zbtb11*$^{+/+}$	*Zbtb11*$^{+/-}$	*Zbtb11*$^{-/-}$
Expected Number	5.25 (25%)	10.5 (50%)	5.25 (25%)
Observed Number	8 (38%)	13 (62%)	0 (0%)

f

Zbtb11$^{+/-}$ X *Zbtb11*$^{+/-}$

↓

Offspring (Total 21 mice)

Sex	Male	Female
Expected Number	10.5 (50%)	10.5 (50%)
Observed Number	10 (48%)	11 (52%)

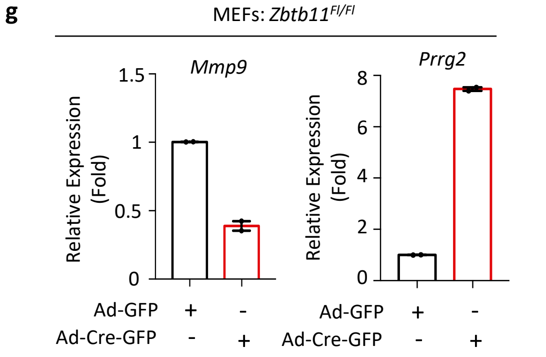

Supplementary Figure 9 Generation and characterization of the Zbtb11 conditional knockout (cKO) mouse model. (**a**) Schematic diagram of the gene targeting strategy for generating the Zbtb11 cKO allele by the CrispR/Cas9 technique. (**b**) Southern blot analysis of the targeted allele of *Zbtb11* in F1 mice. (**c**) Genotyping of F2 mice, revealing wild-type mice and heterozygous or homozygous mice with the floxed *Zbtb11* allele. (**d**) RT–qPCR or RT–hemi-quantitative PCR analysis of *Zbtb11* in *Zbtb11*$^{Fl/Fl}$ MEFs infected with adenovirus expressing GFP or Cre-GFP. Data were shown as the mean ± S.E.M., n=2 experimental replicates. (**e**) The genotypes of the 21 offspring from *Zbtb11*$^{+/-}$ intercrossing. (**f**) The sex of the 21 offspring from *Zbtb11*$^{+/-}$ intercrossing. (**g**) RT–qPCR analysis of *Mmp9* and *Prrg2* expression in *Zbtb11*$^{Fl/Fl}$ MEFs infected with adenovirus expressing GFP or Cre-GFP. Data were shown as the mean ± S.E.M., n=2 experimental replicates. Source data are provided as a Source Data file.

Supplementary Figure 10

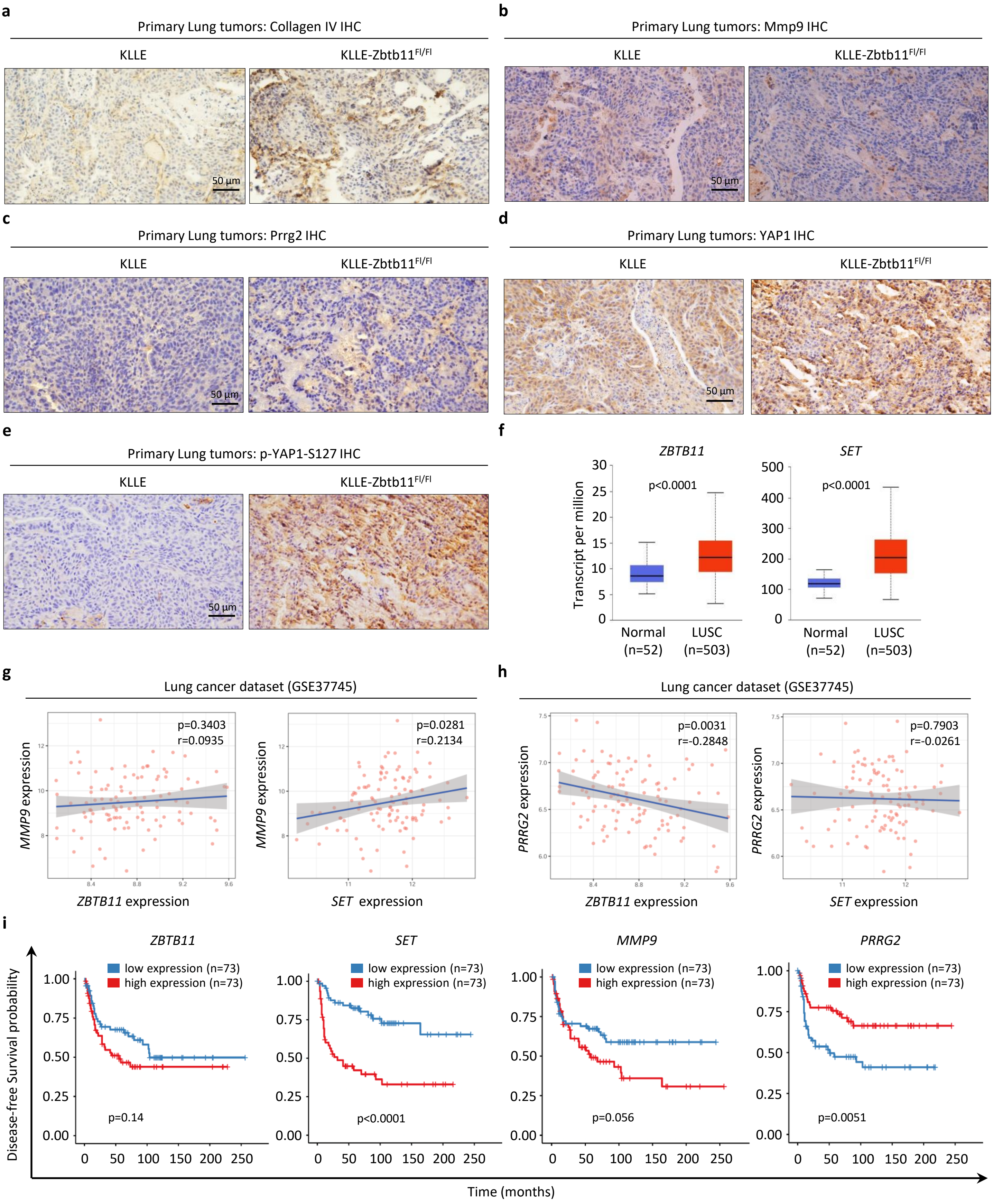

Supplementary Figure 10 Analysis of ZBTB11, SET and their downstream effectors in lung tumors. **(a-e)** Immunohistochemistry (IHC) analysis of Collagen IV **(a)**, Mmp9 **(b)**, Prrg2 **(c)**, YAP1 **(d)**, and p-YAP1-S127 **(e)** in primary lung tumors from KLLE and KLLE-Zbtb11$^{Fl/Fl}$ mice at approximately 9 weeks after Ad-Cre inhalation. **(f)** The positive correlation of high expression of both *ZBTB11* and *SET* with LUSC based on the TCGA database[67]. Data were shown as boxplots with medians, interquartile ranges and lower/upper whiskers in. The p-values were determined by two-sided t-test. **(g)** Correlation analysis of the expression between *MMP9* and *ZBTB11* or *SET* in lung cancer patients, based on GEO dataset (GSE37745)[68]. Pearson's correlation analysis was performed to determine correlation coefficients and p-values. The grey bands represent the 95% confidence interval bands. **(h)** Correlation analysis of the expression between *PRRG2* and *ZBTB11* or *SET* in lung cancer patients, based on GEO dataset (GSE37745)[68]. Pearson's correlation analysis was performed to determine correlation coefficients and p-values. The grey bands represent the 95% confidence interval bands. **(i)** Disease-free Kaplan–Meier plots of lung cancer patients stratified by *ZBTB11*, *SET*, *MMP9* or *PRRG2* expression levels, based on GEO dataset (GSE30219)[69]. The p-values were determined by log-rank test.

Supplementary Figure 11

a

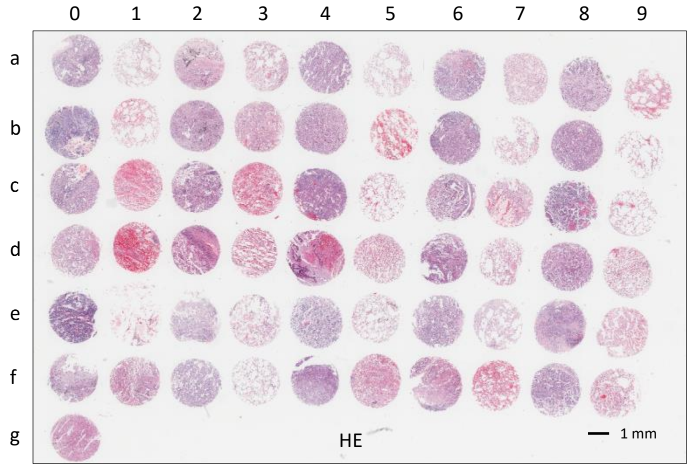

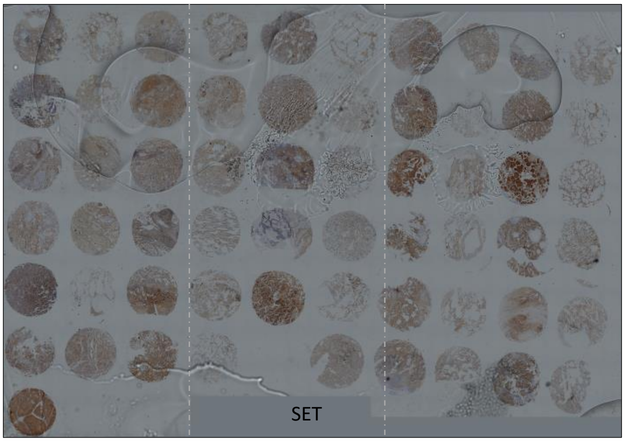

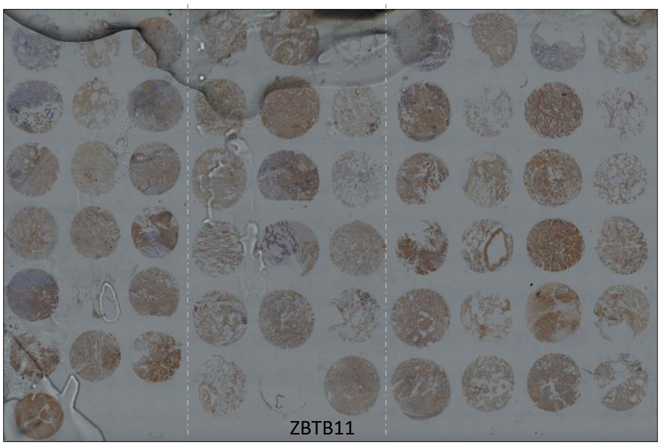

	0	1	2	3	4	5	6	7	8	9
a	T	N	T	N	T	N	T	N	T	N
b	T	N	T	N	T	N	T	N	T	N
c	T	N	T	N	T	N	T	N	T	N
d	T	N	T	N	T	N	T	N	T	N
e	T	N	T	N	T	N	T	N	T	N
f	T	N	T	N	NA	N	T	N	T	N
g	T									

b

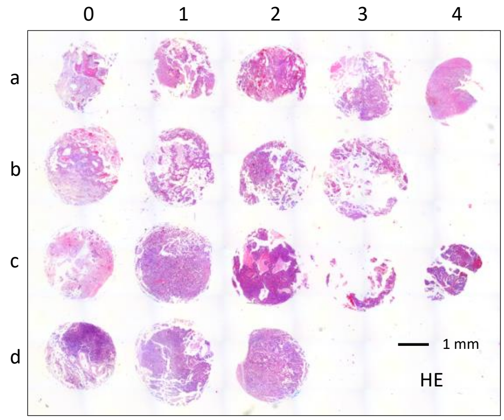

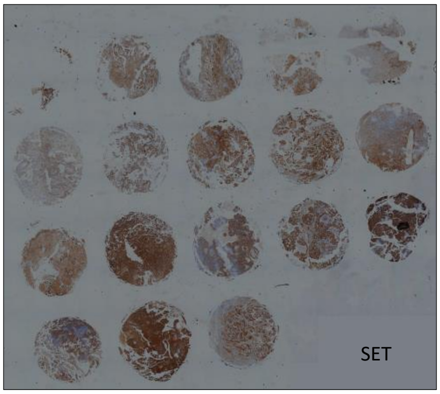

N: Adjacent lung tissue (normal)

T: Primary lung tumor

Metastatic tumor
- mBo: Bone metastasis
- mBr: Brain metastasis
- mAg: Adrenal grand metastasis

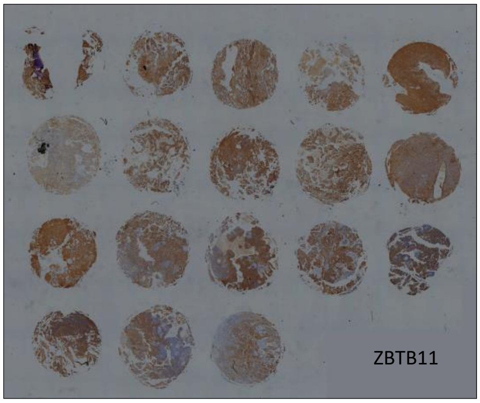

	0	1	2	3	4
a	mBo	mBo	mBr	mBr	mBr
b	mBr	mBr	mBr	mBr	mBr
c	mBr	mBr	mBr	mBr	mBr
d	mBr	mBr	mAg		

Supplementary Figure 11 The expression of SET and ZBTB11 in human lung tumor tissue arrays. (**a**) HE staining and IHC analysis for SET or ZBTB11 in a lung tissue array containing normal lung tissues and adjacent primary tumors (LUAD). (**b**) HE staining and IHC analysis for SET or ZBTB11 in a tissue array containing tumors metastasized from LUAD.
