# Supplementary material for: Oncoprotein SET-associated transcription factor ZBTB11 triggers lung cancer metastasis
Source: Nat Commun. 2024 Feb 14;15:1362. doi: 10.1038/s41467-024-45585-5 (PMC10867109; doi:10.1038/s41467-024-45585-5)
Supplement: Supplementary file 2 — Reporting Summary [file 41467_2024_45585_MOESM2_ESM.pdf]

nature portfolio

Corresponding author(s): Donglai Wang

Last updated by author(s): 2024-1-24

Reporting Summary

Nature Portfolio wishes to improve the reproducibility of the work that we publish. This form provides structure for consistency and transparency in reporting. For further information on Nature Portfolio policies, see our Editorial Policies and the Editorial Policy Checklist.

Statistics

For all statistical analyses, confirm that the following items are present in the figure legend, table legend, main text, or Methods section.

n/a	Confirmed	
☐	☒	The exact sample size (n) for each experimental group/condition, given as a discrete number and unit of measurement
☐	☒	A statement on whether measurements were taken from distinct samples or whether the same sample was measured repeatedly
☐	☒	The statistical test(s) used AND whether they are one- or two-sided<br>*Only common tests should be described solely by name; describe more complex techniques in the Methods section.*
☒	☐	A description of all covariates tested
☐	☒	A description of any assumptions or corrections, such as tests of normality and adjustment for multiple comparisons
☐	☒	A full description of the statistical parameters including central tendency (e.g. means) or other basic estimates (e.g. regression coefficient) AND variation (e.g. standard deviation) or associated estimates of uncertainty (e.g. confidence intervals)
☐	☒	For null hypothesis testing, the test statistic (e.g. F, t, r) with confidence intervals, effect sizes, degrees of freedom and P value noted<br>*Give P values as exact values whenever suitable.*
☒	☐	For Bayesian analysis, information on the choice of priors and Markov chain Monte Carlo settings
☒	☐	For hierarchical and complex designs, identification of the appropriate level for tests and full reporting of outcomes
☐	☒	Estimates of effect sizes (e.g. Cohen's d, Pearson's r), indicating how they were calculated

Our web collection on statistics for biologists contains articles on many of the points above.

Software and code

Policy information about availability of computer code

Data collection

RNA-seq data collection:
H1299 cells were transfected with control siRNA, ZBTB11-specific siRNA and/or SET-specific siRNA twice for a total of 4 days. Each sample group had two biological replicates. Total RNA was extracted using TRIzol® reagent. Before performing RNA-seq analysis, a small aliquot of each sample was analyzed by RT– qPCR to confirm ZBTB11 and SET knockdown efficiency. The RNA quality was assessed by an Agilent 2100 bioanalyzer. The sequencing libraries were generated using the NEBNext® UltraTM RNA Library Prep Kit for Illumina® (NEB, E7530L) following the manufacturer's protocol. Libraries were then sequenced using the Illumina HiSeq platform, and 125 bp/150 bp paired-end reads were generated.

ChIP-seq data collection:
The cells were fixed with 1% formaldehyde, followed by lysis with ChIP lysis buffer (50 mM Tris-HCl pH 8.0, 5 mM EDTA, 1% SDS, 1× protease inhibitor). After sonication, the lysates were centrifuged, and the supernatants were collected and diluted with dilution buffer (20 mM Tris-HCl pH 8.0, 2 mM EDTA, 150 mM NaCl, 1% Triton X-100, 1× protease inhibitor) at a 1:9 ratio. Precleaning of the diluted lysates was performed by salmon sperm DNA saturated protein A agarose
(Millipore, 16-157). The precleaned lysates were incubated with the indicated antibodies overnight, followed by the addition of saturated Protein A agarose for another 2 hr of incubation. The agarose was sequentially washed with TSE I (20 mM Tris-HCl pH 8.0, 2 mM EDTA, 150 mM NaCl, 0.1% SDS, 1% Triton X-100), TSE II (20 mM Tris-HCl pH 8.0, 2 mM EDTA, 500 mM NaCl, 0.1% SDS, 1% Triton X-100), Buffer III (10 mM Tris-HCl pH 8.0, 1 mM EDTA, 0.25 M LiCl, 1% DOC, 1% NP40), and Buffer TE (10 mM Tris-HCl pH 8.0, 1 mM EDTA). The agarose-attached protein–DNA complex was eluted with elution buffer (1% SDS, 0.1 M $NaHCO_3$) and subjected to reverse crosslinking at 65 °C for at least 6 hours. DNA was extracted using a PCR purification kit (Qiagen, 28106). Real-time PCR was performed to detect the relative enrichment of each protein to the indicated genes. The DNA samples were prepared by using a SimpleChIP® Plus Sonication Chromatin IP kit (CST, 56383) according to the manufacturer's protocol. DNA contamination and degradation were checked on agarose gels. The purity of DNA was

evaluated by a NanoPhotometer® spectrophotometer (Implen), and the DNA concentration was measured by a Qubit® dsDNA HS Assay Kit (Thermo, Q32851) in Qubit® 3.0 Fluorometer (Thermo). The library was prepared by using the NEBNext Ultra II DNA Library Prep Kit for Illumina (NEB, E7645) according to the manufacturer's protocol. The quality of the library was assessed by an Agilent Bioanalyzer 2100. Pair-end sequencing of each sample was conducted on an Illumina NovaSeq 6000 platform.

Clinical datasets collection:
The public lung cancer datasets GSE30219 (Rousseaux S et.al, Science Translational Medicine, 2013) and GSE37745 (Botling J et.al, Clinical Cancer Research, 2013) was obtained from Gene Expression Omnibus of NCBI (https://www.ncbi.nlm.nih.gov/geo/). The TCGA-LUAD data was obtained from UALCAN database (https://ualcan.path.uab.edu/analysis.html).

In vitro and In vivo data collection:
Bioluminescent image of Xenograft and transgenic mouse models was collected using Xenogen IVIS spectrum (PerkinElmer, 1400228S). The cell cycle was obtained with the Beckman Coulter CytoPlus C6 platform. The transwell data was photographed by upright microscopy (Leica, DM6 B, Germany). The sections of KLLE mouse lung tissues were photographed by using a ZEISS Axio Scope A1 microscope.

Data analysis

RNA-seq data analysis:
After RNA-seq libraries construction,the index of the reference genome (hg38) was built using HISAT2 v2.0.5, and paired-end clean reads were aligned to the reference genome using HISAT2 v2.0.5. Then, featureCounts v1.5.0-p3 was used to count the read numbers mapped to each gene. Differential expression analysis of the two groups was performed using the DESeq2 R package (1.26.0). DESeq2 provides statistical routines for determining differential expression in digital gene expression data using a model based on the negative binomial distribution. Genes with a p value <0.05 and an absolute value of log2 (fold change)>1 found by DESeq2 were considered differentially expressed. The GO biological process analysis was conducted using the ClusterProfiler R package (4.0.5), and a p value<0.05 was used as the cutoff of statistical significance.

ChIP-seq data analysis:
The raw ChIP-seq reads were aligned to the human reference genome hg38 using Burrows Wheeler Aligner (BWA, v0.7.12). After mapping reads to the reference genome, the model-based analysis of ChIP-seq (MACS, v1.4.2) tools was used for peak calling of IgG- and ZBTB11-binding DNA elements independently under a p value cutoff <0.05 and all other parameters default. Peak annotation was performed using ChIPseeker packages (1.22.1) of R with default parameters, considering the promoter region as 3 kb upstream and 3 kb downstream of the TSS. Finally, we obtained the ZBTB11-specific binding loci by comparing two peak annotation files.

Survival analysis:
survival analysis was conducted by the survival (3.2.7) package of R using lung cancer data from GSE30219 (Rousseaux S et.al, Science Translational Medicine, 2013). According to the quartile of the indicated gene expression level, we divided patients into a high expression group (upper quantile) and a low expression group (lower quantile). Kaplan–Meier survival curves were generated, and the survival impacts of different expression groups were compared by the log-rank test.

Cell cycle analysis:
FlowJo (v10.4) was used to conduct cell cycle analysis.

Transwell and clonal formation data analysis:
JmageJ (v1.8.0) was used to count cell/clone number.

IHC data analysis:
IHC profiler plugin of ImageJ (v1.8.0) was used to quantify IHC data of TMA lung tissues.

Statistical analysis:
The results were presented as the mean ± S.E.M. for bar plot, or as median, interquartile ranges, whiskers for box plot. The statistical significance was determined by using one-sided or two-sided unpaired Student's t test, or two-way ANOVA with Bonferroni's post-hoc test. The correlation test was conducted by the Pearson's correlation method. All statistical analyses were performed using R statistical programming.

The R code used for RNA-seq and ChIP-seq analysis can be accessed at https://github.com/SEO-DataInspire/ZBTB11-SET-project.git.

For manuscripts utilizing custom algorithms or software that are central to the research but not yet described in published literature, software must be made available to editors and reviewers. We strongly encourage code deposition in a community repository (e.g. GitHub). See the Nature Portfolio guidelines for submitting code & software for further information.

Data

Policy information about availability of data

All manuscripts must include a data availability statement. This statement should provide the following information, where applicable:

- Accession codes, unique identifiers, or web links for publicly available datasets
- A description of any restrictions on data availability
- For clinical datasets or third party data, please ensure that the statement adheres to our policy

RNA-seq and ChIP-seq data in this paper have been deposited in NCBI`s Gene Expression Omnibus, and are accessible through GEO Series accession number GSE206957 and GSE206958. The publicly available clinical lung cancer data were available at Gene Expression Omnibus of NCBI (https://www.ncbi.nlm.nih.gov/geo/) under accession number GSE30219 (Rousseaux S et.al, Science Translational Medicine, 2013) and GSE37745 (Botling J et.al, Clinical Cancer Research, 2013) .

The TCGA-LUAD data was obtained from UALCAN database (https://ualcan.path.uab.edu/analysis.html). The remaining data are available within the Article, Supplementary Information or Source Data file. Source data are provided with this paper.

Research involving human participants, their data, or biological material

Policy information about studies with human participants or human data. See also policy information about sex, gender (identity/presentation), and sexual orientation and race, ethnicity and racism.

Reporting on sex and gender	N/A
Reporting on race, ethnicity, or other socially relevant groupings	N/A
Population characteristics	N/A
Recruitment	N/A
Ethics oversight	N/A

Note that full information on the approval of the study protocol must also be provided in the manuscript.

Field-specific reporting

Please select the one below that is the best fit for your research. If you are not sure, read the appropriate sections before making your selection.

[x] Life sciences [ ] Behavioural & social sciences [ ] Ecological, evolutionary & environmental sciences

For a reference copy of the document with all sections, see nature.com/documents/nr-reporting-summary-flat.pdf

Life sciences study design

All studies must disclose on these points even when the disclosure is negative.

Sample size	Sample size was not pre-determined in this study but our sample sizes are consistent with the standard for publications in our filed. The mouse assays included at least 4 or 5 mice per group. The in vitro assays included at least 2 biological replicates. The sample size is indicated in the figure legend for each experiment.
Data exclusions	No data were excluded from the manuscript.
Replication	All results presented in this paper were reliably reproduced. The sample size and number of replicates for each experiment is included in the figure legends. Detailed information of each experiment is also provided in the Methods section.
Randomization	For Xenograft assay, mice were randomly allocated to each experimental group. For transgenic mouse model(KLLE), the two groups of mice being compared each time were from the same parents. For in vitro assay, samples were not randomized as this was not relevant for the individual assay.
Blinding	Investigators were not blinded to the samples when preparing or analyzing the data. Because it was not possible as the data was analyzed by the same individual that performed assays and data collection.

Reporting for specific materials, systems and methods

We require information from authors about some types of materials, experimental systems and methods used in many studies. Here, indicate whether each material, system or method listed is relevant to your study. If you are not sure if a list item applies to your research, read the appropriate section before selecting a response.

Materials & experimental systems

n/a	Involved in the study	
[ ]	[x]	Antibodies
[ ]	[x]	Eukaryotic cell lines
[x]	[ ]	Palaeontology and archaeology
[ ]	[x]	Animals and other organisms
[x]	[ ]	Clinical data
[x]	[ ]	Dual use research of concern
[x]	[ ]	Plants

Methods

n/a	Involved in the study	
[ ]	[x]	ChIP-seq
[ ]	[x]	Flow cytometry
[x]	[ ]	MRI-based neuroimaging

Antibodies

Antibodies used

Antibodies used for Western blot:
ZBTB11 (Bethyl, A303-240A, 1:1000),
SET (Bethyl, A302-262A, 1:1000),
SET (Santa Cruz, sc-133138, 1:1000)
Flag (MBL, PM020, 1:1000),
Myc (Santa Cruz, sc-40, 1:1000),
HA (Roche, 11867423001, 1:2000),
Vinculin (Sigma, V9131, 1:1000),
YAP1 (Santa Cruz, sc-376830, 1:1000),
MMP9(CST, 13667S, 1:1000),
PRRG2 (Abcam, ab228870, 1:1000),
p-YAP1-S127 (ABclonal, AP0489, 1:1000),
p-YAP1-S397 (ABclonal, AP0922, 1:1000),
β-actin (Proteintech, 60008-1-lg, 1:1000),
HDAC1 (Santa Cruz, sc-81598, 1:1000)

Antibodies used for Co-IP:
ZBTB11 (Bethyl: A303-240A, 2μg)
Rabbit IgG (Invitrogen, 10500C, 2μg)
SET (Homemade, 2μg, Co-IP)

Antibodies used for ChIP:
ZBTB11 (Bethyl, A303-240A, 10μg)
SET (Homemade, 10μg)

Antibodies used for ChIP-seq:
ZBTB11 (Bethyl, A303-240A, 10μg)
Rabbit IgG (Invitrogen, 10500C, 2μg)

Antibodies used for IHC:
ZBTB11 (Bethyl, A303-240A, 1:100),
SET (Bethyl, A302-262A, 1:100),
YAP1 (Santa Cruz, sc-376830, 1:100)
Collagen IV (NOVUS, NB120-6586S, 1:100),
MMP9 (ABclonal, A11521, 1:100),
PRRG2 (Abcam, ab228870, 1:100),
p-YAP1-S127 (ABclonal, AP0489, 1:100),

Antibodies used for IF:
ZBTB11 (Bethyl, A303-240A, 1:100)
SET (Sigma, WH0006418M1-100UG, 1:100)

Validation

The homemade SET antibody we used has been validated in previous publications(Donglai Wang et al., 2016). All other antibodies used in this study were purchased from commercial vendors who had validated specificity in human tissues/cells for the specific assays (western blot, immunofluorescence, immunohistochemistry, ChIP, flow cytometry).
Detail information of commercial antibodies can be checked from corresponding websites

ZBTB11: Bethyl, A303-240A, Rabbit Polyclonal, https://fortis-datasheets.s3.us-east-2.amazonaws.com/A303-240A-M-1.pdf
SET: Bethyl, A302-262A, Rabbit Polyclonal, https://fortis-datasheets.s3.us-east-2.amazonaws.com/A302-262A-1.pdf
SET: Santa Cruz, sc-133138, F-9, https://www.scbt.com/p/i2pp2a-antibody-f-9?requestFrom=search
SET: Sigma, WH0006418M1-100UG, Monoclonal, https://www.sigmaaldrich.cn/CN/zh/search/wh0006418m1-100ug?focus=products&page=1&perpage=30&sort=relevance&term=wh0006418m1-100ug&type=product
Rabbit IgG: Invitrogen, 10500C, https://www.thermofisher.cn/cn/zh/antibody/product/Rabbit-IgG-Isotype-Control/10500C
Flag: MBL, PM020, Polyclonal, https://www.mbl-chinawide.cn/uploads/pdf/PM020_ver15.pdf
Myc: Santa Cruz, sc-40, 9E10, https://www.scbt.com/p/c-myc-antibody-9e10?requestFrom=search
HA: Roche, 11867423001, CF10, https://www.sigmaaldrich.cn/CN/zh/search/anti-ha-high-affinity?focus=products&page=1&perpage=30&sort=relevance&term=anti-ha%20high%20affinity&type=product
Vinculin: Sigma, V9131, Monoclonal, https://www.sigmaaldrich.cn/CN/zh/search/v9131?focus=products&page=1&perpage=30&sort=relevance&term=v9131&type=product
YAP1: Santa Cruz, sc-376830, G-6, https://www.scbt.com/p/yap-antibody-g-6?requestFrom=search
Collagen IV: NOVUS, NB120-6586S, Polyclonal, https://www.novusbio.com/products/collagen-iv-antibody_nb120-6586
MMP9: ABclonal, A11521, Primary antibody, https://abclonal.com.cn/catalog/A11521
MMP9: CST, 13667S, D6O3H, https://www.cellsignal.cn/products/primary-antibodies/mmp-9-d6o3h-xp-rabbit-mab/13667
PRRG2: Abcam, ab228870, polyclonal, https://www.abcam.cn/products/primary-antibodies/prrg2-antibody-ab228870.html
p-YAP1-S127: ABclonal, AP0489, Primary antibody, https://abclonal.com.cn/catalog/AP0489

p-YAP1-S397: ABclonal, AP0922, Primary antibody, https://abclonal.com.cn/catalog/AP0922
β-actin: Proteintech, 60008-1-lg, Monoclonal, https://www.ptgcn.com/products/ACTB-Antibody-60008-1-Ig.htm
HDAC1: Santa Cruz, sc-81598, 10E2, https://www.scbt.com/p/hdac1-antibody-10e2?requestFrom=search

Eukaryotic cell lines

Policy information about cell lines and Sex and Gender in Research

Cell line source(s)	NCI-H1299, HEK293T cell lines were obtained from ATCC. NCI-H1299-Luc2-tdT-2 and NCI-H1975 cell lines were obtained from Cell Resource Center of IBMS-CAMS. MEFs cell lines was extracted and primary cultured from transgenic mouse of laboratory.
Authentication	None of cell lines used were directly authenticated after purchase or primary cultured.
Mycoplasma contamination	All cell lines tested negative for mycoplasma contamination.
Commonly misidentified lines (See ICLAC register)	This study did not include any commonly misidentified cell lines.

Animals and other research organisms

Policy information about studies involving animals; ARRIVE guidelines recommended for reporting animal research, and Sex and Gender in Research

Laboratory animals	- Genetically engineered mouse<br>C57BL/6N genetic background Zbtb11-flox mice were generated by BIocytogen Inc. Heterozygous loxP-flanked mice were crossed to generate homozygous loxP-flanked mice and used to get MEF cells.<br><br>For KLLE mice, 129:C57BL/6J background KrasLSL-G12D/+, Lkb1Fl/Fl, and Rosa26-e(CAG-LSL-Luci-EGFP) mice were purchased from Shanghai Model Organisms Center, Inc. KLLE-Zbtb11 mice was obtained by crossing Zbtb11-flox with KLLE mice and enrolled in the experiments at 6~8 weeks of age.<br><br>- Xenograft mouse<br>The 6 weeks old female B-NDG (NSG) mice were purchased from BIocytogen Inc and used in xenograft experiments.<br><br>Mice were housed with up to 5 mice per cage containg clean bedding. A maximal tumor size of 2 cm in any direction and maximal permitted weight loss of 20% was not exceeded in any time point of experiment.
Wild animals	The study did not involve wild animals.
Reporting on sex	No sex-based analysis in this paper.
Field-collected samples	No field-collected samples were used.
Ethics oversight	We submitted the animal experimental ethical inspection (NO: ACUC-A01-2019-014) to Institutional Animal Care and Use Committee (IACUC) of the Chinese Academy of Medical Sciences & Peking Union Medical College (CAMS & PUMC) and received approval. All animal studies, including GEM and xenograft mouse assay, were approved and overseen by Institute of Basic Medical Sciences of the Chinese Academy of Medical Sciences.

Note that full information on the approval of the study protocol must also be provided in the manuscript.

Plants

Seed stocks	*Report on the source of all seed stocks or other plant material used. If applicable, state the seed stock centre and catalogue number. If plant specimens were collected from the field, describe the collection location, date and sampling procedures.*
Novel plant genotypes	*Describe the methods by which all novel plant genotypes were produced. This includes those generated by transgenic approaches, gene editing, chemical/radiation-based mutagenesis and hybridization. For transgenic lines, describe the transformation method, the number of independent lines analyzed and the generation upon which experiments were performed. For gene-edited lines, describe the editor used, the endogenous sequence targeted for editing, the targeting guide RNA sequence (if applicable) and how the editor was applied.*
Authentication	*Describe any authentication procedures for each seed stock used or novel genotype generated. Describe any experiments used to assess the effect of a mutation and, where applicable, how potential secondary effects (e.g. second site T-DNA insertions, mosiacism, off-target gene editing) were examined.*

ChIP-seq

Data deposition

- [x] Confirm that both raw and final processed data have been deposited in a public database such as GEO.
- [x] Confirm that you have deposited or provided access to graph files (e.g. BED files) for the called peaks.

Data access links<br>*May remain private before publication.*	ChIP-seq data (GSE206958) can be obtained from GEO database.
Files in database submission	Processed data files:<br>Input.bw bigWig<br>ZB_IP.bw bigWig<br>IG_IP.bw bigWig<br>ZBTB11_peaks.narrowPeak narrowPeak<br>IgG_peaks.narrowPeak narrowPeak<br><br>Raw files:<br>Input_1.fq.gz fastq<br>Input_2.fq.gz fastq<br>ZB_IP_1.fq.gz fastq<br>ZB_IP_2.fq.gz fastq<br>IG_IP_1.fq.gz fastq<br>IG_IP_2.fq.gz fastq
Genome browser session<br>(e.g. UCSC)	hg38

Methodology

Replicates	None biological replicates were used.
Sequencing depth	Paired end sequencing was performed to obtain 6 GB reads.
Antibodies	ZBTB11 (Bethyl, A303-240A), IgG (CST, 2729S)
Peak calling parameters	Peak calling was conducted by MACS (v1.4.2). The 0.05 was the setting for the p-value option.
Data quality	Details of data analysis and quality assurance are in the Methods section.
Software	Details of the software packages used for ChIP-seq data analysis are in the Methods section.

Flow Cytometry

Plots

Confirm that:

- [x] The axis labels state the marker and fluorochrome used (e.g. CD4-FITC).
- [x] The axis scales are clearly visible. Include numbers along axes only for bottom left plot of group (a 'group' is an analysis of identical markers).
- [x] All plots are contour plots with outliers or pseudocolor plots.
- [x] A numerical value for number of cells or percentage (with statistics) is provided.

Methodology

Sample preparation	The cells were harvested by trypsin digestion and briefly centrifuged. 2e5 cells were fixed with 70% ethanol for 30min. After removal the fix solution and gently washing of the cells with PBS for three times, the cells were stained with 0.5 ml PBS containing 50 "g/ml PI and 200 "g/ml RNase for 30 min at 37 °C.
Instrument	Stained samples were acquired on Beckman Coulter CytoPlus C6 platform.
Software	FlowJo(v10.4) software was used to analyze cell cycle.
Cell population abundance	Cell population abundance was observed during acquiring.

Gating strategy	Staringt from SSC-A/FSC-A, we got live cells, then single cell was obtained by gating FSC-H/FSC-A and SSC-H/SSC-A sequentially. Finaly, the cell cycle was anlysed by FlowJo software.

☒ Tick this box to confirm that a figure exemplifying the gating strategy is provided in the Supplementary Information.
