# Supplementary material for: Oncoprotein SET-associated transcription factor ZBTB11 triggers lung cancer metastasis
Source: Nat Commun. 2024 Feb 14;15:1362. doi: 10.1038/s41467-024-45585-5 (PMC10867109; doi:10.1038/s41467-024-45585-5)
Supplement: Supplementary file 5 — Description of Additional Supplementary Files [file 41467_2024_45585_MOESM5_ESM.pdf]

Title: Supplementary Data file 1
Description: The list of SET-binding proteins identified by MS.

Title: Supplementary Data file 2
Description: The detail information and applications of the antibodies, the siRNA/shRNA/sgRNA and the primers used in this study.
